# Before and after daily stressors: Implications for daily health and well-being in middle-aged and older adults

**DOI:** 10.1093/geroni/igaf122.125

**Published:** 2025-12-31

**Authors:** MacKenzie Hughes, Emily Smith, Shevaun Neupert, Ann Pearman

**Affiliations:** National Opinion Research Center at The University of Chicago, Gig Harbor, Washington, United States; Vermont Department of Health, Burlington, Vermont, United States; North Carolina State University, Raleigh, North Carolina, United States; MetroHealth Medical System, Cleveland, Ohio, United States

## Abstract

Stressor exposure is associated with higher negative affect, worse physical health, and poorer memory performance. However, these processes have typically been evaluated from retrospective accounts of stressors that have already occurred. The goal of the current study was to examine the simultaneous contribution of retrospective daily stressors and forecasts of future stressors as they unfold within the daily lives of middle-aged and older adults. Two hundred sixty-four adults (mean age = 64.11, range 55 - 79) participated in an online daily diary study for 30 consecutive days. Each day they reported on their negative affect as well as challenges with their physical health and memory. They also reported on the stressors encountered in the previous 24 hours and rated the likelihood of experiencing a stressor within the next 24 hours. Separate multilevel models were conducted for each outcome (affect, memory, health) with retrospective stressor exposure and stressor forecasts as within-person predictors. Across the three models, increases in forecasts of future stressors were associated with increases in negative affect, health challenges, and memory issues. However, increases in daily stressor exposure were associated with increases in negative affect and health challenges, but not memory issues. In addition to discussing the significance of these findings, we will explore how understanding this interplay of past daily stressors and appraisals of future stressors may inform targeted interventions designed to support older adults’ well-being.

